# Dual Roles of CD147 in Regulating THP-1 Monocyte Migration and MCP-1-Induced Inflammatory Responses

**DOI:** 10.3390/ijms262210850

**Published:** 2025-11-08

**Authors:** Nutjeera Intasai, Kanokporn Sornsuwan, On-anong Juntit, Thanathat Pamonsupornwichit, Kanyarat Thongheang, Phatcharida Jantaree, Chatchai Tayapiwatana

**Affiliations:** 1 Division of Clinical Microscopy, Department of Medical Technology, Faculty of Associated Medical Sciences, Chiang Mai University, Chiang Mai 50200, Thailand; nutjeera.in@cmu.ac.th; 2 Center of Biomolecular Therapy and Diagnostic, Faculty of Associated Medical Sciences, Chiang Mai University, Chiang Mai 50200, Thailand; kanokporn.sornsuwan@cmu.ac.th (K.S.); onanong.j@cmu.ac.th (O.-a.J.); thanathat.pamon@cmu.ac.th (T.P.); kanyarat_thongheang@cmu.ac.th (K.T.); 3 Office of Research Administration, Chiang Mai University, Chiang Mai 50200, Thailand; 4 Division of Clinical Immunology, Department of Medical Technology, Faculty of Associated Medical Sciences, Chiang Mai University, Chiang Mai 50200, Thailand; 5 Center of Multidisciplinary Technology for Advanced Medicine (CMUTEAM), Faculty of Medicine, Chiang Mai University, Chiang Mai 50200, Thailand; phatcharida.j@cmu.ac.th

**Keywords:** CD147, MCP-1, THP-1, migration, monocyte, inflammatory cytokine

## Abstract

Cluster of Differentiation (CD) 147, a transmembrane glycoprotein, plays a critical role in monocyte function by regulating invasion, migration and cytokine production. This study explored the impact of CD147 on monocyte chemotaxis and inflammatory responses following monocyte chemoattractant protein-1 (MCP-1) modulation using CD147 knockout (CD147^KO^) THP-1 monocytes. CD147^KO^ THP-1 cells exhibited significantly enhanced migration towards MCP-1 and chemoattractants secreted by MDA-MB-231 breast cancer cells compared to wild-type (WT) THP-1 cells, while surface expression of the adhesion molecule CD44 remained unchanged. Despite their increased migration, CD147^KO^ cells showed no significant differences in CC chemokine receptor type 1 (CC1) or CC chemokine receptor type 2 (CCR2) protein expression. Upon MCP-1 stimulation, CD147^KO^ THP-1 monocytes exhibited elevated mRNA expression of *interleukin (IL)-6* and *IL-10*, accompanied by a reduction in *tumor necrosis factor alpha (TNF-α)* at higher MCP-1 concentrations. *IL-6* upregulation in CD147^KO^ THP-1 monocytes appears to be a candidate mediator of their enhanced migratory capacity. In summary, this study highlights the dual role of CD147 as a potential checkpoint in regulating THP-1 monocyte migration, with its function varying depending on the context and microenvironment. Additionally, CD147^KO^ THP-1 monocytes exhibited a shift in the balance between pro- and anti-inflammatory cytokine responses.

## 1. Introduction

CD147, also known as extracellular matrix metalloproteinase inducer (EMMPRIN), is implicated in various aspects of cancer progression [1]. Overexpression of CD147 in cancer cells is associated with tumor invasion [2], metastasis [3], and drug resistance [4]. CD147 is a key regulator in both inflammation and cancer, influencing monocyte migration and extracellular matrix (ECM) remodeling by interacting with molecules critical for chemotaxis, adhesion, and ECM dynamics [5,6,7,8]. Its multifaceted role extends to monocyte migration during inflammation [8] and cancer [5,6]. For instance, stimulating monocytes with atherogenic proinflammatory cytokines leads to CD147 overexpression and matrix metalloproteinase (MMP) activation, resulting in ECM remodeling and monocyte influx and differentiation [7]. Additionally, Cyclophilin A (CyPA), a CD147 ligand, induces monocyte chemotaxis and promotes adhesion to endothelial cells under flow conditions, an effect inhibited by anti-CD147 antibodies [9]. These events are driven by the interaction between CD147 on the cell surface and various molecules within their surrounding microenvironment. Nevertheless, the specific role of CD147 in regulating migration within its host cells remains largely elusive. Among a range of adhesion molecules involved in monocyte migration [10,11], CD44 is of interest in this study. CD44-deficient macrophages have been shown to exhibit reduced motility in transwell migration assays and impaired in vivo migration in response to monocyte chemotactic protein-1 [12]. Based on these findings, the authors concluded that CD44 plays a regulatory role in macrophage recruitment to the lungs during lipopolysaccharide-induced airway inflammation [12].

Monocyte chemoattractant protein-1 (MCP-1/CCL2) is crucial for recruiting monocytes to sites of inflammation or tumors [13,14]. Monocyte chemotaxis is a complex process regulated by various factors, including chemokines, cytokines, adhesion molecules, hypoxia, and components of the extracellular matrix [15,16]. Among these, chemokines and their receptors, such as CCR1 and CCR2, are particularly crucial for immune signaling, operating through specific G protein-coupled receptors [17,18]. MCP-1 primarily binds to CCR2, promoting monocyte motility and migration toward MCP-1 sources [19]. This interaction between CCR2 and MCP-1 guides monocyte recruitment to inflammatory sites [20,21], where they can differentiate into monocytic myeloid-derived suppressor cells (mMDSCs) [22]. The MCP-1/CCR2 signaling axis is a well-characterized mechanism mediating monocyte recruitment and differentiation at inflammatory sites; however, additional molecular pathways governing monocyte migration remain insufficiently defined. Cytokines function in a synergistic manner to further regulate this process. In this context, particular attention is directed to the proinflammatory mediators IL-6 and TNF-α, alongside the anti-inflammatory cytokine IL-10 [23,24]. Elucidating the role of CD147 in modulating monocyte function and regulating inflammatory cytokine gene expression is therefore of considerable significance.

Our recent study revealed that CD147-deficient THP-1 monocytes had a reduced capacity to differentiate into mMDSCs, while CD147-deficient M-LPS THP-1-derived macrophages retained phagocytic activity against Jurkat T cells, with significantly enhanced phagocytosis when treated with the humanized anti-CD147 antibody, HuM6-1B9 [25], which targets CD147. In this study, we aim to further elucidate the role of CD147 in regulating THP-1 monocyte migration and the transcriptional regulation of IL-6, TNF-α, and IL-10, based on their established roles in monocyte-mediating inflammatory responses, with a particular focus on its impact following MCP-1 modulation. These findings will provide deeper insights into the critical processes influenced by CD147, paving the way for future research to extend these investigations in primary monocytes.

## 2. Results

### 2.1. Chemotaxis Migration Assay of WT and CD147^KO^ THP-1 Monocytes

**Figure 1:**
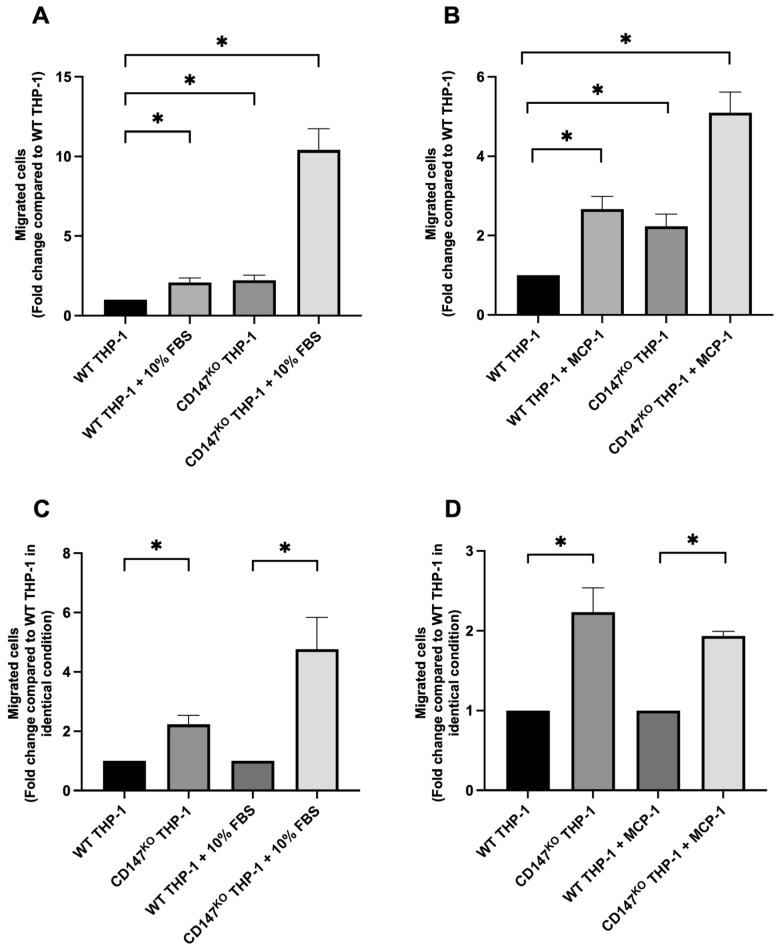
Chemotaxis migration assay of WT THP-1 and CD147^KO^ THP-1 monocytes. Transwell migration assays were conducted to evaluate the chemotactic response of WT and CD147^KO^ THP-1 monocytes in the presence or absence of chemoattractants (10% FBS or MCP-1). Migrated cells were stained with Wright–Giemsa stain and counted under a light microscope using a 20× objective. (**A**,**B**) Relative fold changes in migration of CD147^KO^ THP-1 toward 10% FBS (**A**) and MCP-1 (**B**), normalized to WT THP-1 monocytes. (**C**,**D**) Direct comparison of migratory behavior between WT THP-1 and CD147^KO^ THP-1 monocytes under identical conditions: 10% FBS (**C**) and MCP-1 (**D**). Data are presented as mean ± standard deviation (SD) from three independent experiments. Statistical significance was determined using one-way ANOVA (**A**,**B**) and unpaired *t*-test (**C**,**D**); * *p* < 0.05 indicates statistical significance.

To assess the impact of CD147 deficiency on THP-1 monocyte migration, transwell migration assay was performed using wild-type (WT) and CD147 knockout (CD147^KO^) THP-1 monocytes under basal conditions and in response to specific chemoattractants (Figure S1). Two stimuli were used: 10% FBS, representing a general chemoattractant, and MCP-1, a chemokine enriched in inflammatory and tumor microenvironments. Migration responses were initially normalized to the unstimulated WT control to enable consistent comparison across conditions. Notably, migration of CD147^KO^ THP-1 monocytes was approximately 2-fold higher compared to WT THP-1 monocytes in the absence of chemoattractant (2% FBS, basal condition) (Figure 1). CD147^KO^ THP-1 monocytes exhibited a marked increase in migration, showing a 10-fold increase toward 10% FBS (Figure 1A) and a 5-fold increase toward MCP-1 compared to WT THP-1 monocytes in the absence of chemoattractant (Figure 1B). Under identical conditions, defined as matched media composition, serum concentration, incubation time, and chemokine exposure between genotypes, CD147^KO^ THP-1 monocytes exhibited a 5-fold increase in migration toward 10% FBS (Figure 1C) and a 2-fold increase toward MCP-1 compared to WT THP-1 monocytes (Figure 1D). These findings suggested that CD147 plays a role in THP-1 monocyte migration.

### 2.2. Chemotaxis Migration Assay of WT THP-1 and CD147^KO^ THP-1 Monocytes Toward Chemoattractants Secreted by a Breast Cancer Cell Line, MDA-MB-231

**Figure 2:**
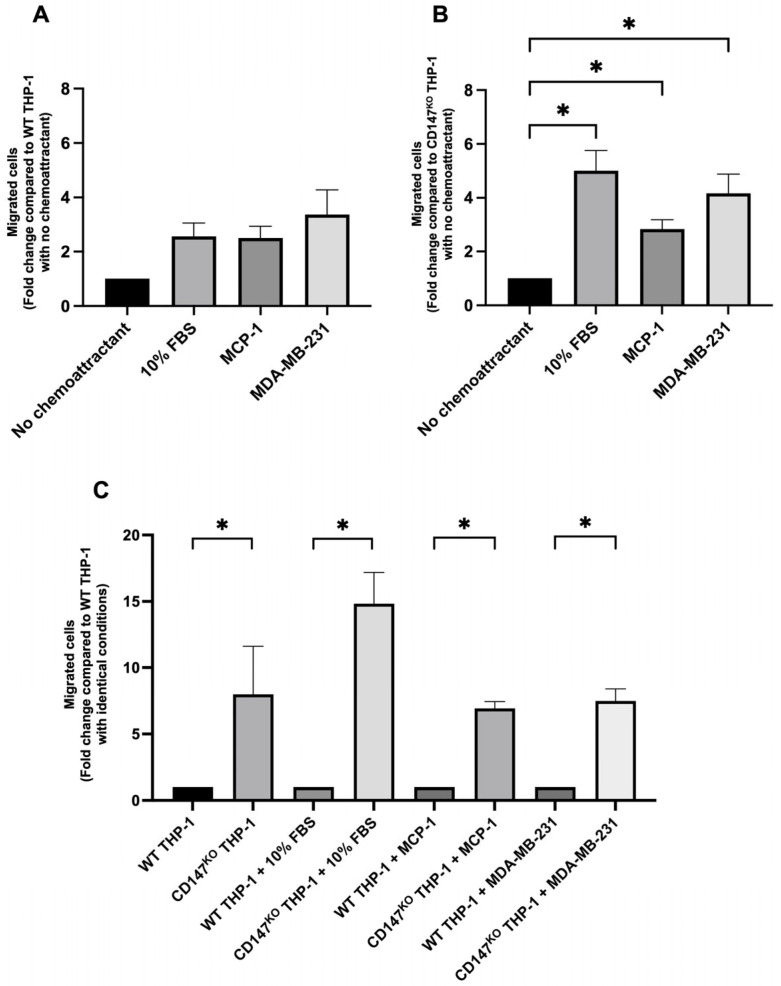
Migration of WT and CD147^KO^ THP-1 monocytes in response to chemoattractants secreted by MDA-MB-231 breast cancer cells. Transwell migration assays were performed to evaluate the migratory response of WT and CD147^KO^ THP-1 monocytes to chemoattractants secreted by MDA-MB-231 cells. 10% FBS-DMEM and 10 ng/mL MCP-1 in 2% FBS-DMEM were used as chemoattractant controls. Migrated cells were stained and counted under a light microscope using a 20× objective. (**A**,**B**) Relative fold changes in migrated cells are shown for WT (**A**) and CD147^KO^ (**B**) THP-1 monocytes, normalized to their respective basal controls (2% FBS-DMEM). (**C**) Direct comparison of migration between WT and CD147^KO^ THP-1 monocytes under identical chemoattractant conditions, including the absence of stimulation. Data represent the mean ± SD from three independent experiments. Statistical analysis was performed using one-way ANOVA for (**A**,**B**) and an unpaired *t*-test (**C**); * *p* < 0.05 indicates statistical significance.

Transwell migration assays were further conducted to explore the chemotaxis migration patterns of WT and CD147^KO^ THP-1 monocytes in response to chemoattractants secreted by a breast cancer cell line, MDA-MB-231 (Figure S2). WT THP-1 monocyte migration toward chemoattractants secreted by MDA-MB-231 cells in 2% FBS-DMEM was approximately 3.4-fold higher than under basal condition (2% FBS-DMEM), whereas migration toward both 10% FBS-DMEM and 10 ng/mL MCP-1 in 2% FBS-DMEM increased by approximately 2.5-fold relative to the basal control. However, these differences were not statistically significant (Figure 2A). In contrast, CD147^KO^ THP-1 monocyte migration toward MDA-MB-231 cell-derived chemoattractants in 2% FBS-DMEM was approximately 4.2-fold higher compared to the basal condition. Migration toward 10% FBS-DMEM and 10 ng/mL MCP-1 in 2% FBS-DMEM was elevated by approximately 5.0-fold and 2.8-fold, respectively, relative to the basal condition. All treatments demonstrated a statistically significant increase in migratory activity compared to the basal condition (Figure 2B). Under identical experimental conditions, CD147^KO^ THP-1 monocytes exhibited significantly higher migratory activity than WT THP-1 monocytes across all chemoattractant conditions, including the absence of chemoattractant (Figure 2C). These findings suggest that CD147 plays a critical role in regulating THP-1 monocyte migration, both intrinsically and in response to various chemoattractants, including those secreted by MDA-MB-231 cells.

### 2.3. Expression of CD147 and CD44 on WT and CD147^KO^ THP-1 Monocytes

**Figure 3:**
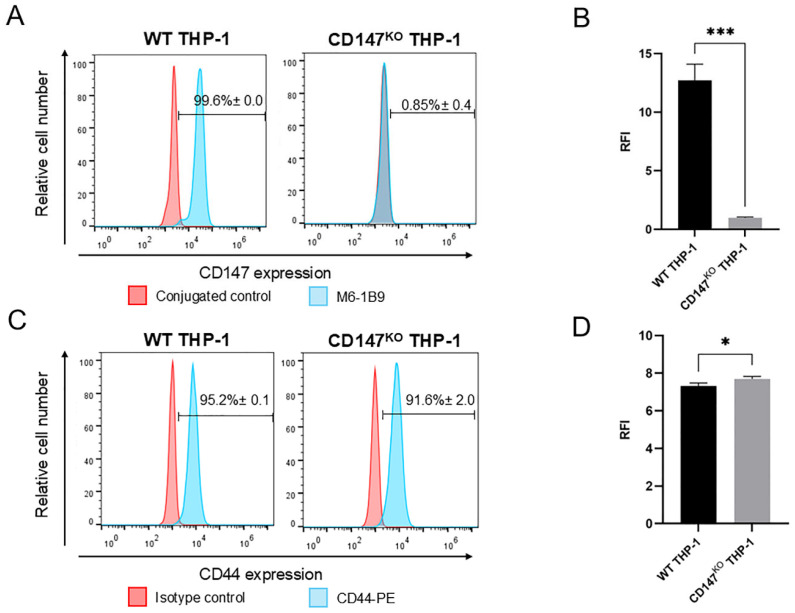
Cell surface expression of CD147 and CD44 on WT and CD147^KO^ THP-1 monocytes. (**A**) Representative overlaid histograms showing CD147 surface expression using mouse anti-CD147 monoclonal antibody, M6-1B9, from three independent experiments. Mean fluorescence intensity (MFI) was analyzed using FlowJo software version 10.0.7, and relative fluorescence intensity (RFI) was calculated as MFI of CD147-stained cells divided by MFI of conjugated control. (**B**) The bar graph represents mean ± SD of CD147 RFI from three independent experiments. (**C**) Representative overlaid histograms showing CD44 surface expression using PE-conjugated mouse anti-human CD44 antibody. (**D**) Bar graph represents the mean ± SD of CD44 RFI from three independent experiments. RFI was calculated as MFI of CD44-stained cells divided by MFI of isotype control. Statistical analysis was performed using unpaired *t*-test. * *p* ≤ 0.05, *** *p* ≤ 0.001.

Flow cytometric analysis confirmed the successful knockout of CD147 on the surface of CD147^KO^ THP-1 monocytes, as indicated by a substantial reduction in both the percentage of CD147 positive cells (Figure 3A) and the relative fluorescence intensity (RFI) compared to WT THP-1 cells (Figure 3B). However, intracellular staining of CD147 revealed that percentage of CD147-positive cells was comparable between WT and CD147^KO^ THP-1 monocytes, whereas the RFI in CD147^KO^ THP-1 monocytes was approximately three-fold lower than WT THP-1 monocytes (Figure S3). In addition to flow cytometric analysis, structured illumination optical sectioning system revealed the intracellular distribution of CD147 in CD147^KO^ THP-1 monocytes relative to the endoplasmic reticulum (ER). In WT cells, CD147 localized predominantly to the perinuclear region (Figure S4A), consistent with normal ER-Golgi trafficking. By contrast, CD147^KO^ cells displayed a markedly altered pattern (Figure S4B), with CD147 signals largely restricted to the cytoplasm and overlapping with the ER marker without clear colocalization, suggesting impaired progression through the secretory pathway. These data align with the flow cytometry results, which showed markedly reduced surface expression of CD147 in CD147^KO^ cells despite retention of intracellular CD147. Furthermore, Western immunoblotting with the MEM-M6/1 antibody [26], which recognizes an epitope in the N-terminal Ig domain (D1) of CD147 detected both the high glycosylated (∼60 kDa) and the low glycosylated (∼36 kDa) forms of CD147 in WT THP-1 monocytes only. These results confirmed the disruption of CD147 expression in CD147^KO^ THP-1 monocytes (Figure S5).

In contrast, the percentage of CD44-positive cells was comparable between WT and CD147^KO^ THP-1 monocytes (Figure 3C), whereas the RFI of CD44 surface expression was slightly increased in CD147^KO^ cells relative to WT cells (Figure 3D). Conversely, intracellular staining of CD44 revealed no notable differences in either the percentage of CD44-positive cells or RFI between WT and CD147^KO^ THP-1 monocytes (Figure S6). These findings suggest that CD147 knockout at the cell surface level does not adversely affect CD44 surface expression in THP-1 monocytes.

### 2.4. Cell Surface and Intracellular Protein Expression of CCR1 and CCR2 in WT and CD147^KO^ THP-1 Monocytes

**Figure 4:**
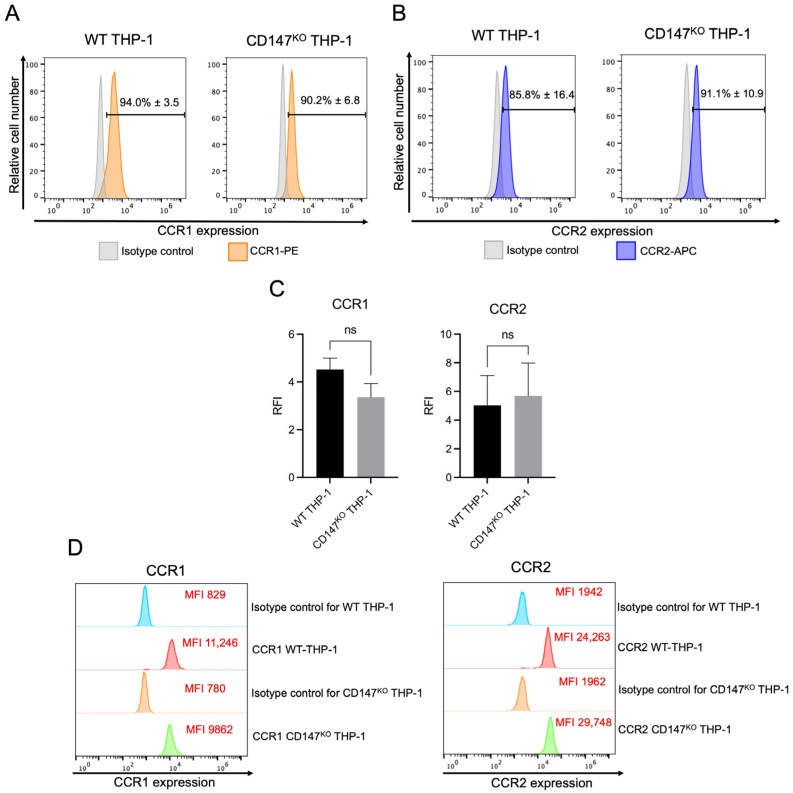
Expression of CCR1 and CCR2 in WT and CD147^KO^ THP-1 monocytes. (**A**) Surface expression of CCR1 in WT and CD147^KO^ THP-1 cells. (**B**) Surface expression of CCR2 in WT and CD147^KO^ THP-1 cells. (**C**) Bar graph depicts the RFI of CCR1 and CCR2 surface expression in WT and CD147^KO^ THP-1 cells. (**D**) Intracellular expression of CCR1 and CCR2 in WT and CD147^KO^ THP-1 cells. (**A**–**C**) Data are presented as mean ± SD from three independent experiments. Statistical analysis was performed using unpaired *t*-test; ns, not significant (*p* ≥ 0.05). (**D**) Representative data from one of three independent experiments.

A comparative analysis of CCR1 and CCR2 expression in WT and CD147^KO^ THP-1 monocytes was performed using flow cytometry. Both cell types exhibited comparable surface expression of CCR1 (Figure 4A) and CCR2 (Figure 4B). RFI analysis revealed no significant differences in CCR1 or CCR2 surface levels between WT and CD147^KO^ THP-1 monocytes, suggesting comparable receptor availability at the cell surface (Figure 4C). Additionally, intracellular levels of CCR1 and CCR2 were comparable between the two groups (Figure 4D). These findings indicate that CD147 deficiency does not alter CCR1 or CCR2 expression in THP-1 monocytes.

### 2.5. Expression of CCR1 and CCR2 mRNA in WT THP-1 and CD147^KO^ THP-1 Monocytes

**Figure 5:**
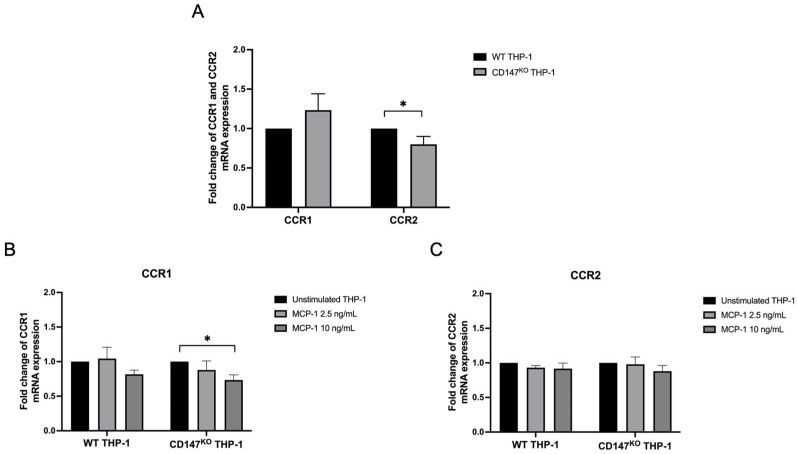
*CCR1* and *CCR2* mRNA expression in WT and CD147^KO^ THP-1 monocytes. (**A**) *CCR1* and *CCR2* mRNA expression in unstimulated CD147^KO^ THP-1 monocytes were compared to those in unstimulated WT THP-1 monocytes. (**B**) *CCR1* and *CCR2* mRNA expression in MCP-1-stimulated WT and CD147^KO^ THP-1 monocytes in comparison to their corresponding unstimulated condition. Fold change in relative gene expression levels was calculated using 2^−ΔΔCt^ method. Data are represented as mean ± SD from triplicate experiments. Statistical significance was determined using (**A**) unpaired *t*-test and (**B**,**C**) one-way ANOVA. * (*p* < 0.05).

WT and CD147^KO^ THP-1 monocytes were treated with or without recombinant human MCP-1/CCL2 at concentrations of 2.5 and 10 ng/mL to investigate the role of CD147 in regulating *CCR1* and *CCR2* mRNA expression using real-time RT-PCR. Under unstimulated conditions, no significant differences in *CCR1* expression were detected, whereas *CCR2* expression was significantly reduced in CD147^KO^ THP-1 monocytes compared to WT (Figure 5A). Upon MCP-1 stimulation, *CCR1* expression remained unchanged in WT THP-1 monocytes but was significantly downregulated in CD147^KO^ THP-1 monocytes at 10 ng/mL relative to their unstimulated condition (Figure 5B). MCP-1 stimulation did not significant alter *CCR2* expression in either cell types (Figure 5C). These results suggest that CD147 deficiency reduces basal *CCR2* mRNA expression and renders *CCR1* mRNA expression in CD147^KO^ THP-1 monocytes more susceptible to downregulation in response to MCP-1 stimulation.

### 2.6. Inflammatory Cytokine Gene Expression on WT and CD147^KO^ THP-1 Monocytes

**Figure 6:**
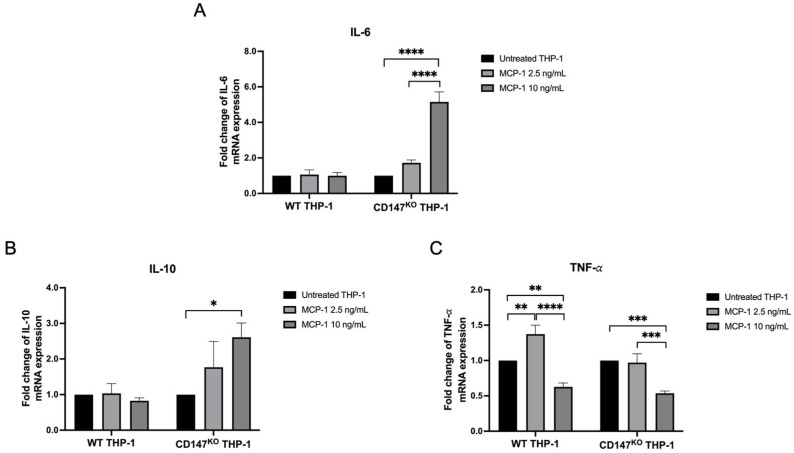
Inflammatory cytokine gene expression on WT and CD147^KO^ THP-1 monocytes. (**A**) *IL-6*, (**B**) *IL-10* and (**C**) *TNF-a* mRNA expression in MCP-1-stimulated WT and CD147^KO^ THP-1 monocytes in comparison to their unstimulated condition. Fold change in relative gene expression levels was calculated using 2^−ΔΔCt^ method. Data are expressed as mean ± SD from triplicate experiments. Statistical significance was determined using one-way ANOVA. * (*p* < 0.05); ** (*p* < 0.01); *** (*p* < 0.001); **** (*p* < 0.0001).

The role of CD147 on *IL-6*, *IL-10* and *TNF-a* mRNA expression of in WT and CD147^KO^ THP-1 monocytes was investigated by real-time RT-PCR. *IL-6* and *IL-10* mRNA expression in CD147^KO^ THP-1 monocytes was upregulated after stimulation with MCP-1, whereas those in WT THP-1 monocytes remained unchanged (Figure 6A and 6B, respectively). *TNF-a* mRNA expression showed a statistically significant increase after stimulation with 2.5 ng/mL MCP-1 in WT THP-1 monocytes. However, a significant reduction in *TNF-a* mRNA expression was observed in both WT and CD147^KO^ THP-1 monocytes following stimulation with 10 ng/mL MCP-1 (Figure 6C).

## 3. Discussion

The mechanisms underlying chemotaxis and the regulation of chemotactic factors involved in monocyte migration, as well as their subsequent tissue infiltration and differentiation into macrophages or dendritic cells, have been extensively studied [27]. Our study was designed to explore the role of CD147 expressed on monocytes, focusing on its impact on chemotaxis and inflammatory cytokine gene expression following MCP-1 modulation employing THP-1 monocytes as a model.

To investigate the functional role of CD147 in monocytes, a CRISPR/Cas9-mediated knockout model was previously established to ablate CD147 surface expression in THP-1 cells [25]. A single guide RNA was designed to target the genomic region immediately upstream of the signal peptide coding sequence in exon 1 of the *BSG* gene [28]. Indel mutations introduced at this site are predicted to disrupt or truncate the signal peptide, thereby impairing its ability to direct nascent CD147 into the endoplasmic reticulum. As a result, CD147 fails to efficiently enter the secretory pathway, leading to a marked reduction in surface expression, as demonstrated by flow cytometry and immunofluorescence analyses. Consistent gene disruption was further supported by Western immunoblotting with the MEM-M6/1 antibody, which recognizes an epitope within the N-terminal Ig domain (D1) of CD147. Because the targeted site lies adjacent to this domain, indel mutations likely altered the structural integrity of the signal peptide–N-terminal region, interfering with epitope recognition and confirming effective disruption of CD147 cell surface expression. These findings imply that CD147 is still synthesized in CD147^KO^ cells but is retained intracellularly, possibly due to misfolding or defective trafficking. Collectively, this evidence indicates that surface depletion of CD147 in CD147^KO^ cells reflects impaired secretion rather than absence of protein expression, reinforcing the utility of this model for dissecting CD147-dependent signaling and membrane-associated functions. Although we did not conduct the CD147^KO^ THP-1 viability assay, the trypan blue dye exclusion assay demonstrated that CD147^KO^ THP-1 cell viability remained consistently above 90% throughout subcultivation, with growth curves similar to those of WT THP-1 cells (unpublished observations).

Migration toward 2% and 10% FBS was used to establish non-chemoattractant and chemoattractant controls, respectively. Because some cell types are highly sensitive to serum deprivation and may undergo apoptosis under serum-free conditions, the use of low serum is preferred for longer assays (>6 h) to reduce cellular stress. Incorporating a low serum concentration in the upper chamber maintains cell viability while establishing an effective chemotactic gradient. Therefore, 2% FBS was used in the upper chamber to balance gradient strength with cell health, the protocol outlined in a previously published study [29]. Notably, the doubling time of the THP-1 cell line is unlikely to have affected the migration results in this study, as the 24-h incubation period falls within the THP-1 replication cycle [30]. Migration responses were initially quantified by normalizing the number of migrated CD147^KO^ cells to the unstimulated WT control, which provided a consistent baseline for assessing the impact of CD147 knockout. Subsequently, direct comparisons between WT and CD147^KO^ THP-1 monocytes under identical conditions allowed a more precise evaluation of each cell type’s intrinsic migratory response to the chemoattractants. CD147 deficiency enhanced THP-1 monocyte migration toward MCP-1 and tumor-derived chemoattractants, suggesting that CD147 negatively regulates both basal and chemotactic migration in THP-1 monocytes. CD147 regulates monocyte migration, with its role varying by context and microenvironment. In rheumatoid arthritis, elevated CD147 expression on monocytes/macrophages enhances MMP secretion and cell invasion [8,9,31]. CyPA is a multifunctional protein that can be secreted and interact with CD147 to promote monocyte chemotaxis under inflammatory conditions. However, CyPA-mediated chemotaxis depends on CD147 as its functional receptor. Extracellular CyPA–induced migration is significantly inhibited by either an anti-CD147 antibody or a CD147 antagonistic peptide, indicating that CD147 is required for CyPA-dependent chemotaxis [9]. Therefore, in CD147^KO^ THP-1 monocytes lacking surface CD147, CyPA cannot exert its chemotactic effect. The enhanced migratory activity observed in CD147^KO^ THP-1 monocytes is thus unlikely to result from CyPA signaling, but rather from compensatory, CyPA-independent remodeling of migratory pathways (e.g., altered MCP-1 responsiveness). As a key adhesion molecule, CD147 also influences cell adhesion dynamics [32]. Some studies suggest that CD147 may restrict monocyte migration under certain conditions, as inhibiting it reduces monocyte adhesion to endothelial cells, indicating its role in both adhesion and migration [32,33]. Additionally, several cell adhesion molecules are known to regulate and restrict cell migration, thereby affecting monocyte mobility [10,11]. Among these, CD44 is a key adhesion molecule involved in monocyte recruitment and is implicated in various cellular processes, including monocyte migration [34,35,36]. It has been reported that CD147, CD44, and EGFR form a complex in MDA-MB-231 cells. The signaling interactions among CD147, hyaluronan–CD44 binding, and the EGFR–Ras–ERK pathway are known to regulate the invasive properties of breast epithelial cells. Based on these findings, we hypothesized that CD147 might influence CD44 surface expression on THP-1 monocytes and potentially contribute to their migratory capacity. However, CD44 expression was comparable between WT and CD147^KO^ THP-1 monocytes, suggesting that CD147 deficiency does not affect CD44 expression. Taken together, the enhanced migration observed in CD147^KO^ THP-1 monocytes in response to chemoattractants, including MCP-1 and those secreted by the MDA-MB-231 cells, suggests that CD147 typically functions to restrict THP-1 monocyte migration.

The chemokine receptors CCR1 and CCR2 are well-recognized markers of monocyte migration [37]. To investigate the role of CD147 in regulating chemokine receptors, we compared the expression of CCR1 and CCR2 in both WT and CD147^KO^ THP-1 monocytes. The mRNA and protein expression levels of CCR1 were comparable between the two groups. In contrast, the mRNA expression of *CCR2* in CD147^KO^ THP-1 monocytes was reduced, while its protein expression remained consistent across both groups. Steady-state surface CCR2 reflects synthesis, recycling, and ligand-induced internalization [38,39,40,41]; thus, reduced CCR2 transcripts can coexist with near-constant surface CCR2 at baseline. Thus, knocking out CD147 did not alter CCR1 or CCR2 protein expression in THP-1 monocytes. Furthermore, the enhanced migration of CD147^KO^ monocytes in response to MCP-1 was not associated with changes in these receptors, except for an increase in *CCR1* mRNA expression at a concentration of 10 ng/mL MCP-1. MCP-1 signals primarily via CCR2, with additional handling by atypical receptors (ACKR1, ACKR2) [42,43]. Accordingly, our interpretation centers on CCR2—dependent migration and receptor dynamics, although other chemokine receptors beyond CCR1 and CCR2 may also contribute to the migratory behavior of CD147^KO^ THP-1 monocytes. Nevertheless, MCP-1–induced CCR1/CCR2 modulation cannot be excluded and will be investigated in future work.

Additionally, emerging evidence suggests that MCP-1 functions may extend beyond chemotaxis, influencing monocyte behaviors such as maturation, differentiation and cytokine production [23,44]. Given this broader role, we aimed to investigate how CD147 modulates inflammatory cytokine expression in responses to MCP-1 stimulation. Specifically, our study focused on comparing the cytokine response profiles of wild-type and CD147^KO^ THP-1 monocytes to elucidate the functional role of CD147 in MCP-1-mediated signaling. Our objective is not to delineate the full temporal dynamics of cytokine induction but rather to assess whether CD147 deficiency alters the magnitude of MCP-1–induced responses. While ELISA quantifies secreted proteins and provides functional insights, analyzing mRNA expression is crucial for understanding CD147-driven transcriptional regulation. mRNA levels reflect gene regulation, the primary focus of our study. RT-PCR is more sensitive than ELISA, as it can detect even small amounts of mRNA, while secreted cytokines often require concentration for detection. Gene expression analysis offers higher sensitivity and allows for the early detection of cytokine changes, which may be missed at the protein level due to biological or technical limitations [45]. Moreover, changes in secreted cytokines at the picomolar level are hardly detected. Therefore, gene expression analysis offers greater sensitivity and is more effective for early cytokine detection. We further investigate the impacts of CD147 on the transcriptional regulation of *IL-6*, *IL-10* and *TNF-α* based on their established roles in monocyte-mediating inflammatory responses, with a particular focus on its impact following MCP-1 modulation. The 18-h time point was selected based on multiple reports indicating that cytokine gene expression in response to MCP-1 or similar inflammatory stimuli peaks between 16–24 h [46,47,48]. Upon stimulation with 10 ng/mL MCP-1, CD147^KO^ THP-1 monocytes exhibited an increase in mRNA expression of the pro-inflammatory cytokine *IL-6*, balanced by a decrease in *TNF-α* and an increase in the anti-inflammatory cytokine *IL-10*, maintaining a response similar to that of WT THP-1 monocytes. While IL-10 is classically regarded as immunosuppressive, its effects are context-dependent. It has been reported to have immunostimulatory roles under certain conditions, such as enhancing effector T-cell responses in inflammatory settings [49]. In CD147^KO^ THP-1 monocytes, the shift toward increased *IL-6* and *IL-10* expression, along with decreased *TNF-α* expression, likely reflects a compensatory mechanism rather than a shift toward suppressive phenotypes. This pronounced cytokine upregulation in CD147^KO^ cells directly supports our central hypothesis that CD147 modulates monocyte inflammatory responses to MCP-1. Interestingly, although CD147 knockout did not alter CD44 expression in THP-1 cells, it significantly upregulated *IL-6* expression. IL-6 secreted by CD147-deficient monocytes may enhance monocyte migration through both autocrine and paracrine mechanisms. In the autocrine context, the secreted IL-6 may directly increase the migratory capacity of monocytes. IL-6 has been reported to induce MCP-1 expression in peripheral blood mononuclear cells (PBMCs) and the U937 monocytic cell line, highlighting the role of IL-6 in monocyte recruitment [50]. In the paracrine context, the secreted IL-6 may stimulate MDA-MB-231 to increase MCP-1 expression, as reported by Weng et al. [51], thereby reinforcing a chemotactic loop that further promotes monocyte recruitment. Additionally, TNF signaling has been implicated in driving the accumulation of MDSCs [52]. In our study, the significant reduction in *TNF-α* expression in CD147^KO^ THP-1 monocytes upon MCP-1 stimulation may counteract the suppressive effects of increased *IL-10*, supporting our hypothesis that these cells are less likely to differentiate into MDSCs. We recently reported that CD147^KO^ THP-1 monocytes did not affect *TNF-a* and *IL-10* mRNA expression following stimulation with phorbol myristate acetate (PMA), either in combination with LPS (M-LPS) or IL-4 (M-IL-4) [25]. This finding is consistent with results from a study by Moller A. et al. in monocytic ER-Hoxb8-derived cells [53]. Thus, this information supports the role of CD147 in regulating cytokine expression in monocytes, with its function varying based on the context and microenvironment.

## 4. Materials and Methods

### 4.1. Cell Lines

Wild-type (WT) THP-1 (TIB-202^TM^) (ATCC, Manassas, VA, USA) and CD147 knockout (CD147^KO^) THP-1 monocytes were cultured in Roswell Park Memorial Institute 1640 (RPMI-1640) medium (Gibco, Thermo Fisher Scientific, Waltham, MA, USA) supplemented with 10% FBS (Gibco, Thermo Fisher Scientific, Waltham, MA, USA), 100 U/mL penicillin (Gibco, Thermo Fisher Scientific, Waltham, MA, USA), 100 μg/mL streptomycin (Gibco, Thermo Fisher Scientific, Waltham, MA, USA) and 2 mM L-glutamine (Gibco, Thermo Fisher Scientific, Waltham, MA, USA). MDA-MB-231 (HTB-26^TM^) (ATCC, Manassas, VA, USA) was cultured in Dulbecco’s Modified Eagle Medium (DMEM) (Gibco, Thermo Fisher Scientific, Waltham, MA, USA) with the same supplements.

CD147^KO^ THP-1 cells, generated using a CD147-targeting single guide RNA (sgRNA) [28] as described in our previous study [25], were further investigated in this study. The sgRNA sequence 5′-GCGAGGAATAGGAATCATGG-3′ was designed to target a region immediately upstream of the CD147 (BSG) signal peptide [28]. Briefly, a ribonucleoprotein (RNP) complex was assembled using spCas9 (Integrated DNA Technologies, Coralville, IA, USA) and a sgRNA targeting CD147. The RNP complex was then transfected into 5 × 10^5^ THP-1 cells using the 4D-Nucleofector™ X Kit S (Lonza, Basel, Switzerland) with the FF100 program of the 4D-Nucleofector™ X unit. After nucleofection, the cells were cultured in RPMI-1640 medium supplemented with 20% FBS.

### 4.2. Transwell Migration Assay

A transwell migration assay was conducted to assess the migratory behavior of WT and CD147^KO^ THP-1 monocytes using 24-well transwell inserts with 3 μm pore size (NEST Scientific, Woodbridge, NJ, USA) as described in previously published studies [29,54,55,56]. WT or CD147^KO^ THP-1 monocytes with 3 × 10^5^ cells in 250 μL of 2% FBS-RPMI-1640 were loaded into the upper chamber of the transwell insert. Six hundred and fifty microliters of either 10% FBS-RPMI-1640 or 10 ng/mL recombinant human MCP-1/CCL2 protein (Sinobiological, Beijing, China) in 2% FBS-RPMI-1640 was added to the lower chamber as chemoattractants. RPMI-1640 containing 2% FBS was used as a non-chemoattractant control. The cells were then incubated at 37 °C and 5% CO_2_ for 24 h. The number of cells that migrated across the membrane to the lower chamber was stained with Wright–Giemsa and counted under light microscope. The results are presented as the fold change in the number of migrated cells relative to their respective controls.

MDA-MB-231 cells secrete MCP-1, along with other chemokines that may synergize to promote THP-1 migration [57]. To investigate the migration of CD147^KO^ THP-1 toward chemoattractants secreted by a triple negative breast cancer cell line, MDA-MB-231, 2 × 10^5^ cells of MDA-MB-231 in 650 mL of 2% FBS-DMEM was added to the lower chamber and cultured overnight. The other conditions were carried out as previously described, with the exception that all experiments were conducted in DMEM rather than RPMI-1640, including both WT and CD147^KO^ THP-1 monocytes.

### 4.3. Flow Cytometric Analysis for Expression of CD147, CD44, CCR1 and CCR2

To evaluate CD147 surface expression on WT and CD147^KO^ THP-1 cells, the cells were stained as previously described [25]. In brief, the cells were incubated with 10% human AB serum in FACS buffer on ice for 30 min and subsequently stained with mouse anti-CD147 (M6-1B9) mAb. FITC-conjugated F(ab’)2 goat anti-mouse IgG+IgM (H+L) (Immunotools, Friesoythe, Germany) was employed as secondary antibody. CD147 expression of the stained cells was assessed using BD Accuri C6 (BD BioSciences, Franklin Lakes, NJ, USA) and analyzed using FlowJo software version 10.0.7.

Cell surface expression of CD44, CCR1 and CCR2 on WT and CD147^KO^ THP-1 cells was assessed. WT and CD147^KO^ THP-1 cells were harvested and incubated with 10% human AB serum in a FACS buffer on ice for 30 min to block Fc receptors. Subsequently, cells were stained with PE-conjugated anti-human CD44 antibody (mouse IgG1 isotype) (Immunotools, Friesoythe, Germany), PE-conjugated anti-human CD191 (CCR1) antibody (mouse IgG1 isotype) (Biolegend, San Diego, CA, USA) or APC-conjugated anti-human CD192 (CCR2) antibody (mouse IgG2a isotype) (Biolegend, San Diego, CA, USA) at 1:10 dilution. PE-conjugated mouse IgG1 antibody (Immunotools, Friesoythe, Germany), or APC-conjugated mouse IgG2a antibody (Immunotools, Friesoythe, Germany) were used as isotype-matched controls. Finally, the cells were washed three times with a FACS buffer and subsequently fixed with 1% paraformaldehyde in phosphate-buffered saline (PBS). The stained cells were determined using BD Accuri C6 (BD BioSciences, Franklin Lakes, NJ, USA) and analyzed using FlowJo software version 10.0.7. For comparing the expression level of CCR1 and CCR2, the RFI was calculated.

For intracellular staining of CCR1 and CCR2, WT and CD147^KO^ THP-1 cells were fixed with 4% paraformaldehyde in PBS for 15 min at room temperature. Cells were subsequently washed twice with a FACs buffer and permeabilized with PBS containing 0.1% saponin, 5% FBS and 0.1% sodium azide. The cellular Fc receptors were blocked with 10% human AB serum in PBS containing 0.1% saponin, 5% FBS and 0.1% sodium azide on ice for 30 min. The cells were then stained with PE-conjugated anti-human CD191 (CCR1) antibody or APC-conjugated anti-human CD192 (CCR2) antibody at 1:10 dilution and incubated on ice for 30 min. The same isotype-matched controls, as described above, were used. After twice washing with PBS containing 0.01% saponin, 5% FBS and 0.1% sodium azide, the stained cells were analyzed using the same instrument and software.

### 4.4. Real-Time RT-PCR

Real-time reverse transcription polymerase chain reaction (real-time RT-PCR) was utilized to analyze *CCR1*, *CCR2*, *IL-6*, *IL-10* and *TNF-a* gene expression. WT and CD147^KO^ THP-1 monocytes at 1.5 × 10^6^ cells were treated with or without recombinant human MCP-1/CCL2 protein at 2.5 and 10 ng/mL in 2% FBS-RPMI-1640 medium for 18 h. Total RNA was isolated from the treated or untreated WT and CD147^KO^ THP-1 cells using the RNeasy Mini Kit (QIAGEN, Germantown, MD, USA), followed by conversion to cDNA through the SuperScript™ III First-Strand Synthetic System (ThermoFisher Scientific, Waltham, MA, USA). Fifty nanograms of cDNA served as the template for determining the mRNA expression of *CCR1*, *CCR2*, *IL-6*, *IL-10* and *TNF-a*. *GAPDH* was used as an internal control. The relative fold gene expression of the treated or untreated WT and CD147^KO^ THP-1 cells was calculated using the 2^−ΔΔCt^ method. Primer pairs used are shown in Table 1.

## 5. Conclusions

Our findings identify CD147 as a checkpoint in THP-1 monocyte migration and immune response modulation. Knocking down CD147 could enhance immune cell infiltration into tumors, thereby improving the effectiveness of antitumor response. However, the potential involvement of chemokine receptors other than CCR1 and CCR2, or cell adhesion molecules beyond CD44, in regulating the migration of CD147^KO^ THP-1 monocytes cannot be ruled out. Therefore, this study lays the groundwork for further functional studies, such as transcriptomic analyses, to elucidate the signaling pathways underlying MCP-1-mediated migration in CD147^KO^ THP-1 monocytes. Additionally, the altered cytokine profile in CD147^KO^ THP-1 monocytes, marked by increased *IL-6* and *IL-10* and reduced *TNF-α*, suggests that CD147 influences the balance between pro- and anti-inflammatory responses. This could be exploited to reshape the tumor microenvironment by reducing the differentiation of immunosuppressive mMDSCs while promoting pro-inflammatory M1 macrophage polarization as shown in our recent study [25]. Notably, IL-6 secreted by CD147-deficient monocytes may promote monocyte migration through both autocrine and paracrine mechanisms. However, as our dataset reflects transcriptional changes, we have outlined protein-level validation (e.g., IL-6/IL-10 ELISA and transwell compartment sampling) as a priority for future work to substantiate the proposed IL-6–driven autocrine/paracrine mechanism underlying the enhanced migratory capacity of CD147^KO^ THP-1 monocytes. Finally, although the THP-1 cell line is commonly used as a model for studying monocyte and macrophage biology, it may not fully replicate the biological functions and stimulus responses observed in primary monocytes [59]. Therefore, the role of CD147 in the migration of primary monocytes and macrophages will be further explored in future studies.

## Figures and Tables

**Table 1:** Primer sequences [58].

Gene	Forward Primer 5′-3′	Reverse Primer 5′-3′
*CCR1*	TGCTCTGCTCACACTCATGG	TCCAAAGCTGTCCGTTTGAT
*CCR2*	TGACAGGCACAGATGAATGG	ATCATCTCCTGGCTGAATGC
*IL-6*	AGACAGCCACTCACCTCTTC	AGTGCCTCTTTGCTGCTTTC
*IL-10*	CCTGCCTAACATGCTTCGAG	GGCAACCCAGGTAACCCTTA
*TNF-α*	CTGCACTTTGGAGTGATCGG	TACAACATGGGCTACAGGCT
*GAPDH*	ACCCAGAAGACTGTGGATGG	CAGCTCAGGGATGACCTTGG

## Data Availability

The original contributions presented in this study are included in the article/Supplementary Material. Further inquiries can be directed to the corresponding author.

## Supplementary Materials

The following supporting information can be downloaded at: https://www.mdpi.com/article/10.3390/ijms262210850/s1.
