# Supplementary material for: Dual Roles of CD147 in Regulating THP-1 Monocyte Migration and MCP-1-Induced Inflammatory Responses
Source: Int J Mol Sci. 2025 Nov 8;26(22):10850. doi: 10.3390/ijms262210850 (PMC12652673; doi:10.3390/ijms262210850)
Supplement: Supplementary file 1 [file ijms-26-10850-s001.zip › ijms-3951748_Supplementary figures.pdf]

Supplementary figures

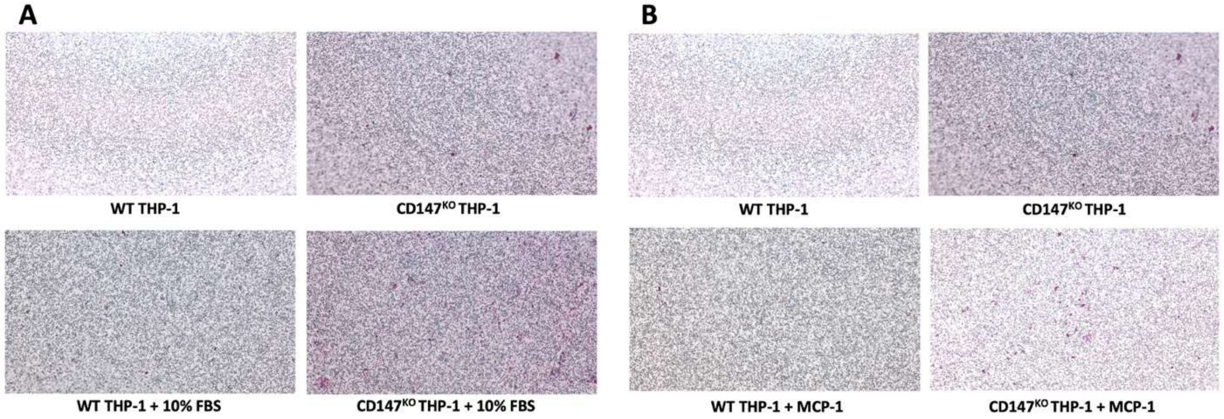

Figure S1. Chemotaxis migration assay of WT THP-1 and CD147^{KO} THP-1 monocytes. Transwell migration assays were conducted to evaluate the chemotactic response of WT and CD147^{KO} THP-1 monocytes in the absence (upper panel) or presence (lower panel) of chemoattractants, (A) 10% FBS-RPMI 1640 or (B) 10 ng/mL MCP-1 in 2% FBS-RPMI 1640. Migrated cells were stained with Wright-Giemsa stain and counted under a light microscope using a 20× objective.

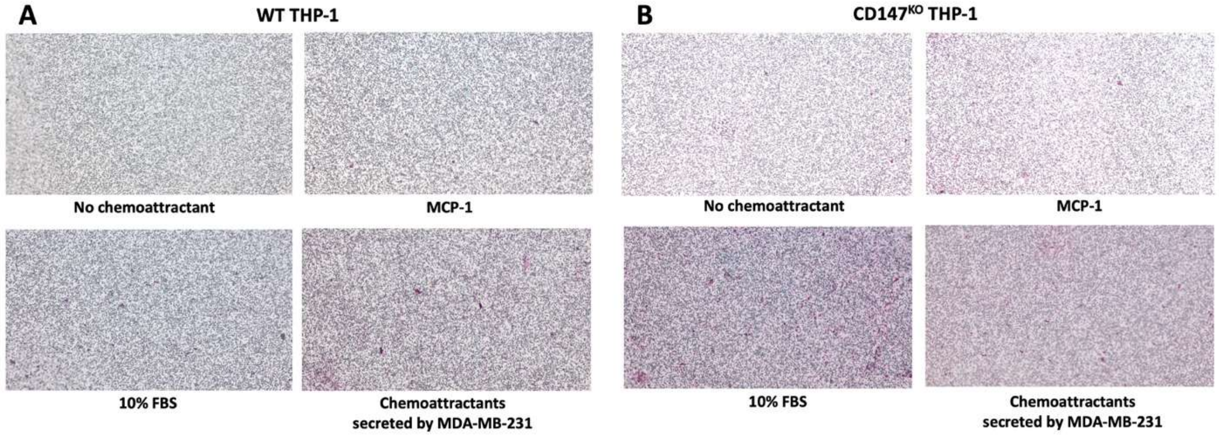

Figure S2. Migration of WT and CD147^{KO} THP-1 monocytes in response to chemoattractants secreted by MDA-MB-231 breast cancer cells. Transwell migration assays were performed to evaluate the migratory response of (A) WT and (B) CD147^{KO} THP-1 monocytes. The culture of MDA-MB-231 cells in 2% FBS-DMEM (lower right) was used as a source of tumor-derived chemoattractants. 2% FBS-DMEM (upper left) served as a non-chemoattractant control. As chemoattractant controls, 10 ng/mL MCP-1 in 2% FBS-DMEM (upper right) and 10% FBS-DMEM (lower left) were used. Migrated cells were stained with Wright-Giemsa stain and counted under a light microscope using a 20× objective.

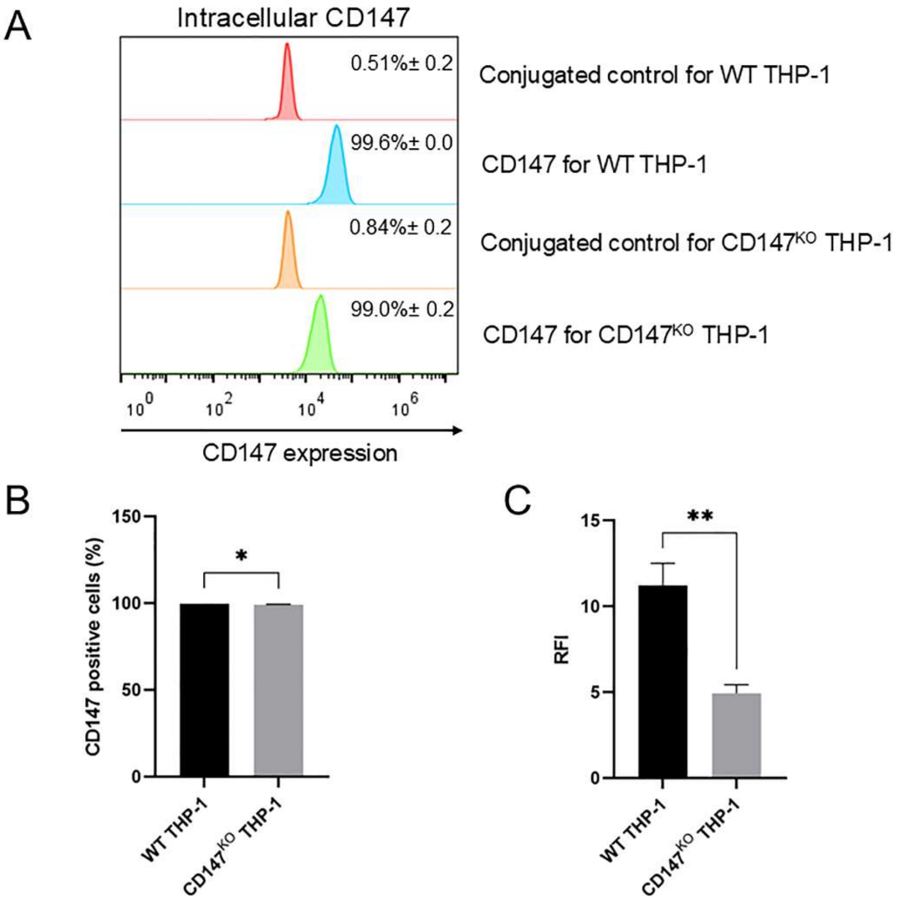

Figure S3. Intracellular CD147 expression in WT and $CD147^{KO}$ THP-1 monocytes. WT and $CD147^{KO}$ THP-1 monocytes were stained intracellularly with mouse anti-CD147 mAb (M6-1B9), followed by FITC-conjugated $F(ab')_2$ goat anti-mouse IgG+IgM (H+L). (A) Representative histogram from one of three replicate experiments. Bar graphs showed mean ± SD of (B) percentage of CD147 positive cells and (C) RFI. Statistical analysis was performed using unpaired *t-test*. * ($p \leq 0.05$), ** ($p \leq 0.01$).

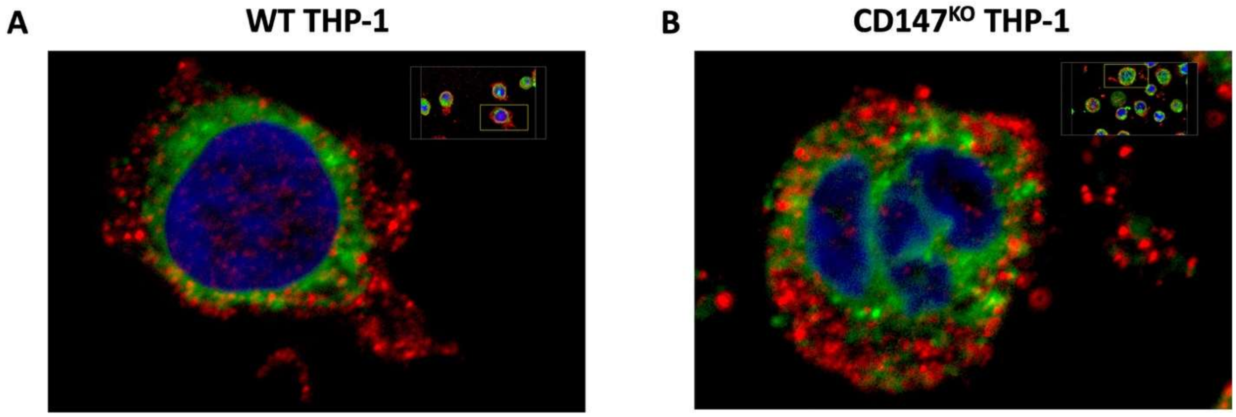

Figure S4. Subcellular localization of CD147 in WT and CD147^{KO} THP-1 monocytes. WT and CD147^{KO} THP-1 cells were fixed, permeabilized, and stained for CD147 (red), the endoplasmic reticulum (ER, green), and nuclei (blue). (A) In WT THP-1 cells, CD147 localizes to the perinuclear region, consistent with its trafficking through the ER-Golgi secretory pathway toward the plasma membrane. (B) In CD147^{KO} THP-1 cells, impaired entry of CD147 into the secretory pathway leads to its retention and accumulation within the cell. Insets show full-field views with the analyzed regions highlighted (yellow boxes). Images were acquired using Apotome structured illumination microscopy.

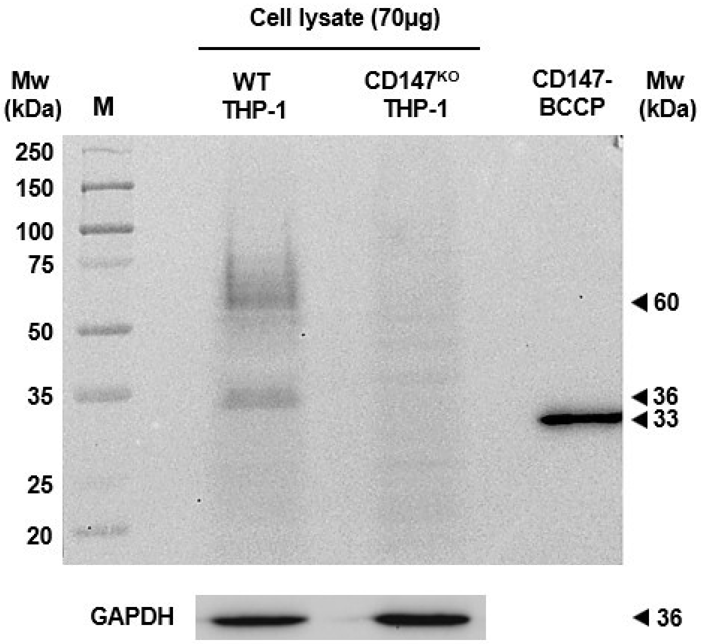

Figure S5. Western blot analysis of CD147 in WT and $CD147^{KO}$ THP-1 monocytes. Whole-cell lysates (70 µg) from WT and $CD147^{KO}$ THP-1 monocytes were separated on a 10% SDS-PAGE and transferred to a nitrocellulose membrane. Purified CD147-BCCP served as a positive control for CD147 detection. In WT THP-1 lysates, CD147 was observed as two prominent bands corresponding to the high glycosylated (~60 kDa) and the low glycosylated (~36 kDa) forms, whereas no CD147 signal was detected in $CD147^{KO}$ THP-1 lysates. GAPDH served as a loading control. M, protein standard.

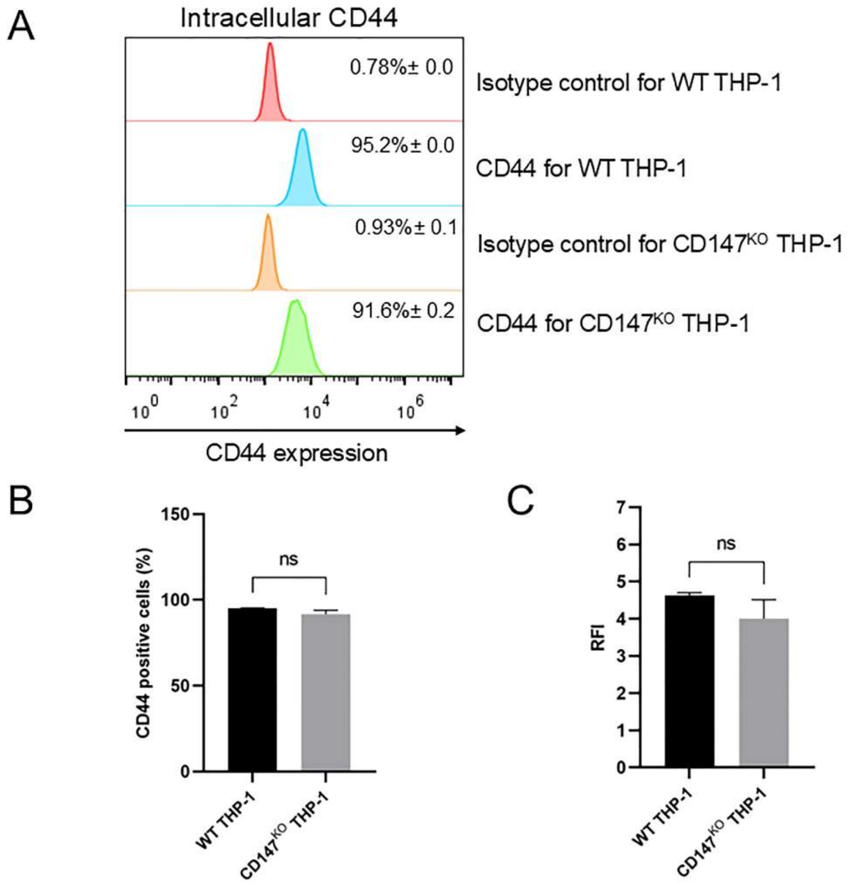

Figure S6. Intracellular CD44 expression in WT and CD147^{KO} THP-1 monocytes. WT and CD147^{KO} THP-1 monocytes were stained intracellularly with PE-conjugated mouse anti-human CD44 antibody. (A) Representative histogram from one of three replicate experiments. Bar graphs showed mean ± SD of (B) percentage of CD44 positive cells and (C) RFI. Statistical analysis was performed using unpaired *t-test*. ns ($p \geq 0.05$), not statistically significant difference.
