# Supplementary material for: Dual Roles of CD147 in Regulating THP-1 Monocyte Migration and MCP-1-Induced Inflammatory Responses
Source: Int J Mol Sci. 2025 Nov 8;26(22):10850. doi: 10.3390/ijms262210850 (PMC12652673; doi:10.3390/ijms262210850)
Supplement: Supplementary file 1 [file ijms-26-10850-s001.zip › ijms-3951748_Supplementary methods.pdf]

Supplementary methods

Intracellular CD147 and CD44 staining in WT and CD147^{KO} THP-1 monocytes

WT and CD147^{KO} THP-1 cells (5×10^5 cells) were fixed with 4% paraformaldehyde in PBS for 15 min at room temperature. Cells were subsequently washed with FACs buffer twice and permeabilized with PBS containing 0.1% saponin, 5% FBS and 0.1% sodium azide. Fc receptors were blocked with 10% human AB serum in PBS containing 0.1% saponin, 5% FBS and 0.1% sodium azide on ice for 30 min. For intracellular CD147 staining, cells were stained with mouse anti-CD147 mAb (M6-1B9). After washing with FACs buffer, FITC-conjugated F(ab′)2 goat anti-mouse IgG+IgM (H+L) (Immunotools, Friesoythe, Germany) was employed as secondary antibody. For intracellular CD44 staining, cells were stained with PE-conjugated mouse anti-CD44 antibody (Immunotools, Friesoythe, Germany). PE-conjugated mouse IgG1 antibody was served as an isotype-matched control. Intracellular expression of CD147 and CD44 was assessed using a BD Accuri C6 flow cytometer (BD Biosciences, NJ, USA). Data were analyzed using FlowJo software (Tree Star Inc., Ashland, OR, USA).

Intracellular localization of CD147 in WT and CD147^{KO} THP-1 monocytes

CD147^{KO} THP-1 monocytes were seeded onto 96-well glass-bottom plates pre-coated with 50 µg/mL poly-L-lysine (100 µL/well, 5 min, room temperature [RT]), followed by three washes with distilled water and air drying. Cells (4×10^4/well), resuspended in serum-free RPMI-1640, were seeded and incubated at 37 °C for 15 min to allow attachment. Fixation was performed with 3.2% paraformaldehyde for 20 min at RT, followed by washing with HBSS. The cells were then permeabilized with 0.25% Triton X-100 for 15 min at RT and washed again with HBSS. For staining, cells were incubated at RT in the dark for 30 min with Hoechst 33342 (5 µg/mL) and PhenoVue™ Fluor 488-Concanavalin A (100 µg/mL) from the PhenoVue™ Cell Painting Kit (Revvity, Waltham, MA, USA), followed by HBSS washes. Fc receptor blocking was performed using 10% FBS in PBS for 30 min at RT. Cells were incubated then with 10 µg/mL humanized anti-CD147 antibody (HuM6-1B9) [23] for 30 min at RT, washed with PBS, and subsequently incubated with rabbit anti-human IgG-DyLight 650 (1:250 dilution) for 30 min at RT. After final washes with HBSS, 200 µL of HBSS was added to each well. Plates were sealed with adhesive foil and stored at 4 °C in the dark until imaging using a Zeiss Axio Observer 7 Inverted Microscope equipped with Apotome.2 (Carl Zeiss Microscopy, LLC., NY, USA).

Western immunoblotting of CD147 in WT and CD147^{KO} THP-1 monocytes

Five-million WT or CD147^{KO} THP-1 cells were washed twice with PBS. Cell pellets were lysed with RIPA buffer supplemented with protease inhibitor cocktail III (Merck, DA, DE) on ice for 15 minutes.

Lysates were clarified by centrifugation at 15,000 × g for 15 min at 4 °C and total protein was quantified by BCA assay (Thermo Fisher Scientific, Waltham, MA, USA). Seventy-micrograms of whole-cell lysates were separated on a 10% SDS-PAGE and transferred to nitrocellulose membrane. After membrane blocking with 5% BSA (Merck, DA, DE) in TBS-T (0.1% Tween 20 in PBS), membrane was incubated with 5 μg/mL of mouse anti-CD147 antibody (MEM-M6/1) (Abcam, Cambridge, UK) diluted in 2% BSA in TBS-T at 4 °C for overnight. Membrane was washed three times with TBS-T, followed by incubating with HRP-conjugated goat anti-mouse Igs (1:2,500 dilution) (Seracare, UK) at room temperature for 1 hour. Excess secondary antibody was removed through three consecutive washes with TBS-T. The reaction was developed using KPL LumiGlo® chemiluminescent substrate (Seracare, UK). Image was analyzed using Bio-rad Image Lab software (Bio-rad, CA, USA). The molecular weight of the high glycosylated (HG; ~60 kDa) and the low glycosylated (LG; ~36 kDa) forms of CD147 was verified using Precission Plus Protein™ Standards Kaleidoscope™ (Bio-rad, CA, USA). Recombinant CD147-BCCP was included as a positive control, while GAPDH was employed as a loading control in this experiment. Membrane was incubated in antibody stripling buffer at RT for 20 minutes. After washing three times with TBS-T, membrane was incubated with 5% skimmed-milk in TBS-T for 1 hour. The membrane HRP-conjugated mouse anti-human GAPDH antibody (Biolegend, CA, USA) (diluted to 1:1000 in 2% skimmed-milk in TBS-T) was added to membranes, followed by 1 hour incubation. After washing step, the reaction was developed using KPL LumiGlo® chemiluminescent substrate (Seracare, UK).
